# Sex-specific transcriptional rewiring in the brain of Alzheimer’s disease patients

**DOI:** 10.3389/fnagi.2022.1009368

**Published:** 2022-10-31

**Authors:** Jose A. Santiago, James P. Quinn, Judith A. Potashkin

**Affiliations:** ^1^NeuroHub Analytics, LLC, Chicago, IL, United States; ^2^Q Regulating Systems, LLC, Gurnee, IL, United States; ^3^Cellular and Molecular Pharmacology Department, Center for Neurodegenerative Diseases and Therapeutics, The Chicago Medical School, Rosalind Franklin University of Medicine and Science, North Chicago, IL, United States

**Keywords:** Alzheimer’s disease, co-expression networks, sex differences, switch genes, asymptomatic Alzheimer’s disease

## Abstract

Sex-specific differences may contribute to Alzheimer’s disease (AD) development. AD is more prevalent in women worldwide, and female sex has been suggested as a disease risk factor. Nevertheless, the molecular mechanisms underlying sex-biased differences in AD remain poorly characterized. To this end, we analyzed the transcriptional changes in the entorhinal cortex of symptomatic and asymptomatic AD patients stratified by sex. Co-expression network analysis implemented by SWItchMiner software identified sex-specific signatures of switch genes responsible for drastic transcriptional changes in the brain of AD and asymptomatic AD individuals. Pathway analysis of the switch genes revealed that morphine addiction, retrograde endocannabinoid signaling, and autophagy are associated with both females with AD (F-AD) and males with (M-AD). In contrast, nicotine addiction, cell adhesion molecules, oxytocin signaling, adipocytokine signaling, prolactin signaling, and alcoholism are uniquely associated with M-AD. Similarly, some of the unique pathways associated with F-AD switch genes are viral myocarditis, Hippo signaling pathway, endometrial cancer, insulin signaling, and PI3K-AKT signaling. Together these results reveal that there are many sex-specific pathways that may lead to AD. Approximately 20–30% of the elderly have an accumulation of amyloid beta in the brain, but show no cognitive deficit. Asymptomatic females (F-asymAD) and males (M-asymAD) both shared dysregulation of endocytosis. In contrast, pathways uniquely associated with F-asymAD switch genes are insulin secretion, progesterone-mediated oocyte maturation, axon guidance, renal cell carcinoma, and ErbB signaling pathway. Similarly, pathways uniquely associated with M-asymAD switch genes are fluid shear stress and atherosclerosis, FcγR mediated phagocytosis, and proteoglycans in cancer. These results reveal for the first time unique pathways associated with either disease progression or cognitive resilience in asymptomatic individuals. Additionally, we identified numerous sex-specific transcription factors and potential neurotoxic chemicals that may be involved in the pathogenesis of AD. Together these results reveal likely molecular drivers of sex differences in the brain of AD patients. Future molecular studies dissecting the functional role of these switch genes in driving sex differences in AD are warranted.

## Introduction

Sex disparities have been reported in numerous preclinical, epidemiological, and clinical studies on Alzheimer’s disease (AD), the most common cause of dementia worldwide. According to the Alzheimer’s Disease Association, approximately two-thirds of Americans with AD are women (Rajan et al., 2021). Earlier epidemiological studies reported that older women have a higher risk of developing dementia than men (Fratiglioni et al., 1997; Ott et al., 1998; Andersen et al., 1999; Letenneur et al., 1999; Ruitenberg et al., 2001; Di Carlo et al., 2002). Nonetheless, most of these studies were from European countries. Indeed, epidemiological studies from North and South America have not observed a significant sex correlation with AD (Bachman et al., 1993; Rocca et al., 1998). One study showed a higher incidence of AD among men (Ganguli et al., 2000). Several investigations have suggested that the higher incidence of AD among women is due to the longer life expectancy rather than sex-specific factors (Hebert et al., 2001). Recent epidemiological studies, however, suggest that sex and gender differences in the risk of AD may be influenced by geographical regions (Mielke et al., 2022).

Animal and molecular studies have also revealed sex correlations with AD. Sex-specific differences in AD have been associated with diet, metabolic factors, inflammation, and comorbidities. For example, a high-fat diet elicited a greater metabolic impairment, visceral fat accumulation, and glucose intolerance in female but not in male 3x-Tg AD mice (Gannon et al., 2022). Similarly, male ApoE knock-in mice but not females exposed to a high-fat diet showed markers of chronic neuroinflammation and liver dysfunction (Mattar et al., 2022). ApoE knock-in female mice exposed to the same diet displayed spatial learning and memory impairments without the neuroinflammation or liver dysfunction observed in males. In addition to sex differences, these studies highlight the complex interaction between genetic and environmental factors in AD.

Several potential mechanisms have been posited to explain sex differences in AD, including hormonal regulation, physiological differences, and sex chromosomes. Recent investigations show that women with AD exhibit greater cognitive resilience, verbal memory reserve, and preservation of brain structure when exposed to pathological tau (Digma et al., 2020; Ossenkoppele et al., 2020). Furthermore, increased expression of X chromosome genes is associated with slower cognitive decline in women. In contrast, some X chromosome genes are associated with neuropathological tau burden in men but not in women (Davis et al., 2021). Sex hormone signaling is another strong hypothesis supporting a sex-specific vulnerability in AD. Several studies have shown a higher incidence of AD in women after menopause (Fisher et al., 2018). Recently, inhibition of follicle-stimulating hormone (FSH) signaling improved cognition in mice with AD (Xiong et al., 2022).

Unbiased bioinformatic approaches have unveiled sex-specific differences in AD studies. For example, an analysis of blood transcriptomic profiles from women with advanced AD identified the PI3K-AKT signaling, estrogen, and atherosclerosis as shared dysregulated pathways in diabetes (Santiago et al., 2019). Sex-stratified single-cell gene and pathway analysis revealed opposite transcriptional changes in the entorhinal cortex of males and females with AD (Belonwu et al., 2022).

Recently, a new network-based methodology called SWItchMiner has enabled the analysis of co-expression networks and the identification of key genes known as ‘switch genes’ that may play a crucial role in phenotypic transitions. Switch genes are associated with drastic transcriptional changes and may play a critical role in disease pathogenesis. This bioinformatic method has successfully identified switch genes in numerous biological settings, including cancer (Fiscon et al., 2018b,c; Falcone et al., 2019), chronic obstructive pulmonary disease (Paci et al., 2020), physical activity (Santiago et al., 2022), AD (Potashkin et al., 2019; Bottero et al., 2021), frontotemporal and vascular dementias (Potashkin et al., 2020), and amyotrophic lateral sclerosis (Santiago et al., 2021; Bottero et al., 2022). Further, sex-specific switch genes were identified in the blood of ALS patients (Santiago et al., 2021). Although switch genes have been identified in AD (Potashkin et al., 2019; Bottero et al., 2021), analysis of co-expression networks and switch genes stratified by sex has not been explored.

Here we built on previous work and investigated sex-specific transcriptional changes in the entorhinal cortex of AD patients. Imaging studies have revealed that the entorhinal cortex, a brain region important in memory formation and learning, is one of the first regions affected in AD (Khan et al., 2014). The analysis was performed on subjects stratified by sex with symptomatic AD and those with intact cognition but neuropathological findings consistent with AD (asymAD). Including asymAD individuals in this study is important given that approximately 20–30% of the aging population with preserved cognition have an accumulation of amyloid beta (Rodrigue et al., 2009). The investigation of this phenotype is expected to reveal pathways associated with disease progression or cognitive resilience.

## Materials and methods

### Demographic and clinical information of study subjects

GSE118553 microarray was accessed from the NCBI GEO database. This transcriptomic study contained 78 brain samples from the entorhinal cortex from 16 controls (sex (M/F, 9/7), 28 asymAD (M/F, 8/20), and 34 AD subjects (M/F, 13/21). These samples were obtained from the Medical Research Council London Neurodegenerative Diseases Brain Bank. The mean age (±SD) of subjects was: controls: 71.9 (15.6), asymAD: 85.4 (9.5), and AD: 83.9 (9.7). Neuropathology was assessed using the BRAAK staging. Braak staging (±SD) of subjects was: controls:0, AsymAD: 2.2 (1.2), and AD: 4.9 (1). The disease duration was 11.8 (5.8) for AD subjects. Control subjects were classified as healthy without dementia or neuropathological evidence of AD. AsymAD subjects had no clinical sign of dementia at the time of death but showed the presence of AD neuropathology. AD cases were clinically diagnosed and positive for neuropathological features consistent with AD. No information about comorbidities or medication usage was available in the original study. All other clinical information about the study participants is published elsewhere in (Patel et al., 2019).

### Swim analysis to identify switch genes

Raw data from GSE118553 was imported into SWIM (Paci et al., 2017). The SWIM algorithm consists of several steps described in detail elsewhere (Paci et al., 2017; Fiscon et al., 2018a). Genes with no or low expression were removed in the preprocessing stage. The fold changes were set for each array in the filtering step, and genes that were not significantly expressed between cases compared to controls were removed. SWIM analysis was performed using the following comparisons: F-AD vs. controls, F-AD vs. F-asymAD, M-AD vs. controls, and M-AD vs. M-asymAD. The fold changes used in this study were 1.5 for F-AD vs. controls, M-AD vs. controls, male AD vs. M-asymAD, and 1.4 for F-AD vs. F-asymAD. The False Discovery Rate method (FDR) was used for multiple test corrections. Pearson correlation analysis was performed to build a co-expression network of genes differentially expressed between cases and controls. The k-means algorithm was used to identify communities within the network. Using the clusterphobic coefficient Kπ and the global-within module degree Zg, a heat map was created. The coefficient *Kπ* measures the external and internal node connections, whereas *Zg* measures the extent each node is connected to others in its community. A node is classified as a hub when Zg >5. The average Pearson correlation coefficient (APCC) between the expression profile of each node and its nearest neighbors is used to build the heat map. Using the APCC, three types of hubs are defined; date hubs that show low positive co-expression with their partners (low APCC), party hubs that show high positive co-expression (high APCC), and nodes that have negative APCC values are called fight-club hubs. In the final step, switch genes are identified and defined as not being a hub in their cluster (low *Zg* < 2.5), having many links outside their group (*Kπ* > 0.8, when *Kπ* is close to 1, most of its links are external to its module), and having a negative average weight of incident links (APCC <0). Switch genes interact outside their community, are not in local hubs, and are mainly anti-correlated with their interaction partners.

### Pathway analysis of switch genes

Biological and functional analysis of switch genes was performed using NetworkAnalyst and ExpressAnalyst (Xia et al., 2014).^1^ Official gene symbols of switch genes for each dataset F-AD, F-asymAD, M-AD, and M-asymAD were imported into NetworkAnalyst and analyzed separately. Functional enrichment analysis was performed using the KEGG database. Bipartite networks were visualized within NetworkAnalyst and ExpressAnalyst interfaces. Venn diagram analysis was used to identify shared and unique pathways.

### Transcription factor analysis of switch genes

Gene-transcription factor analysis was performed in NetworkAnalyst. Switch genes obtained from F-AD, F-asymAD, M-AD, and M-asymAD were analyzed separately. Transcription factor and gene target data were derived from the Encyclopedia of DNA Elements (ENCODE). ENCODE uses the BETA Minus Algorithm in which only peak intensity signal <500 and the predicted regulatory potential score < 1 are used. Transcription factors were ranked according to network topology measurements, including degree and betweenness centrality. Venn diagram analysis was used to identify shared and unique transcription factors.

### Protein-chemical interaction analysis

Switch genes from the different datasets, F-AD, F-asymAD, M-AD, and M-asymAD, were imported into NetworkAnalyst for protein-chemical interaction analysis. NetworkAnalyst uses the chemicals and drug data from the Comparative Toxicogenomics Database (CTD). Chemicals were ranked according to the degree and betweenness centrality.

## Results

### Identification of switch genes in the entorhinal cortex of AD patients stratified by sex

**Figure 1:**
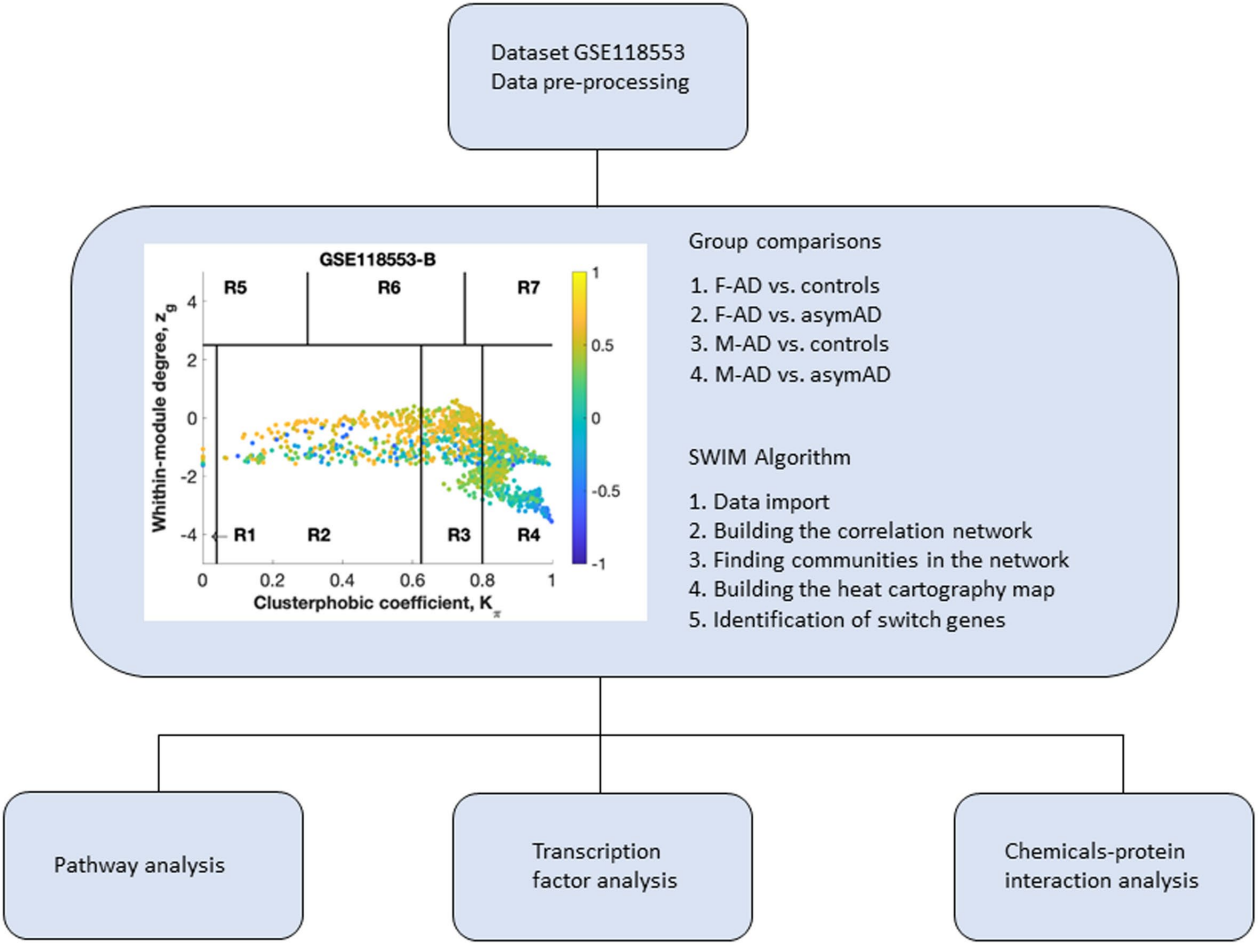
Overall study design. Dataset 118,553 containing transcriptomic data from the entorhinal cortex of Alzheimer’s disease (AD) individuals was imported into SWIM software for co-expression network analysis. Samples from symptomatic and asymptomatic AD individuals were stratified by sex and analyzed separately. Switch genes identified in males and females were analyzed further for pathway analysis, transcription factors, and chemical interactions. The switch genes are denoted by the blue nodes falling in the region R4. F-AD, females with AD; F-asymAD, asymptomatic AD females; M-AD, males with AD; M-asymAD, asymptomatic AD males.

To identify key genes that may reveal sex-specific molecular mechanisms in the entorhinal cortex of AD, we performed a co-expression network analysis of AD samples stratified by sex using SWIM software on the dataset GSE118553. Switch genes were identified using the following comparisons: F-AD vs. controls, F-AD vs. F-asymAD, M-AD vs. controls, and M-AD vs. M-asymAD, hereafter referred to as F-AD, F-asymAD, M-AD, and M-asymAD switch genes. The overall study workflow is presented in Figure 1.

**Figure 2:**
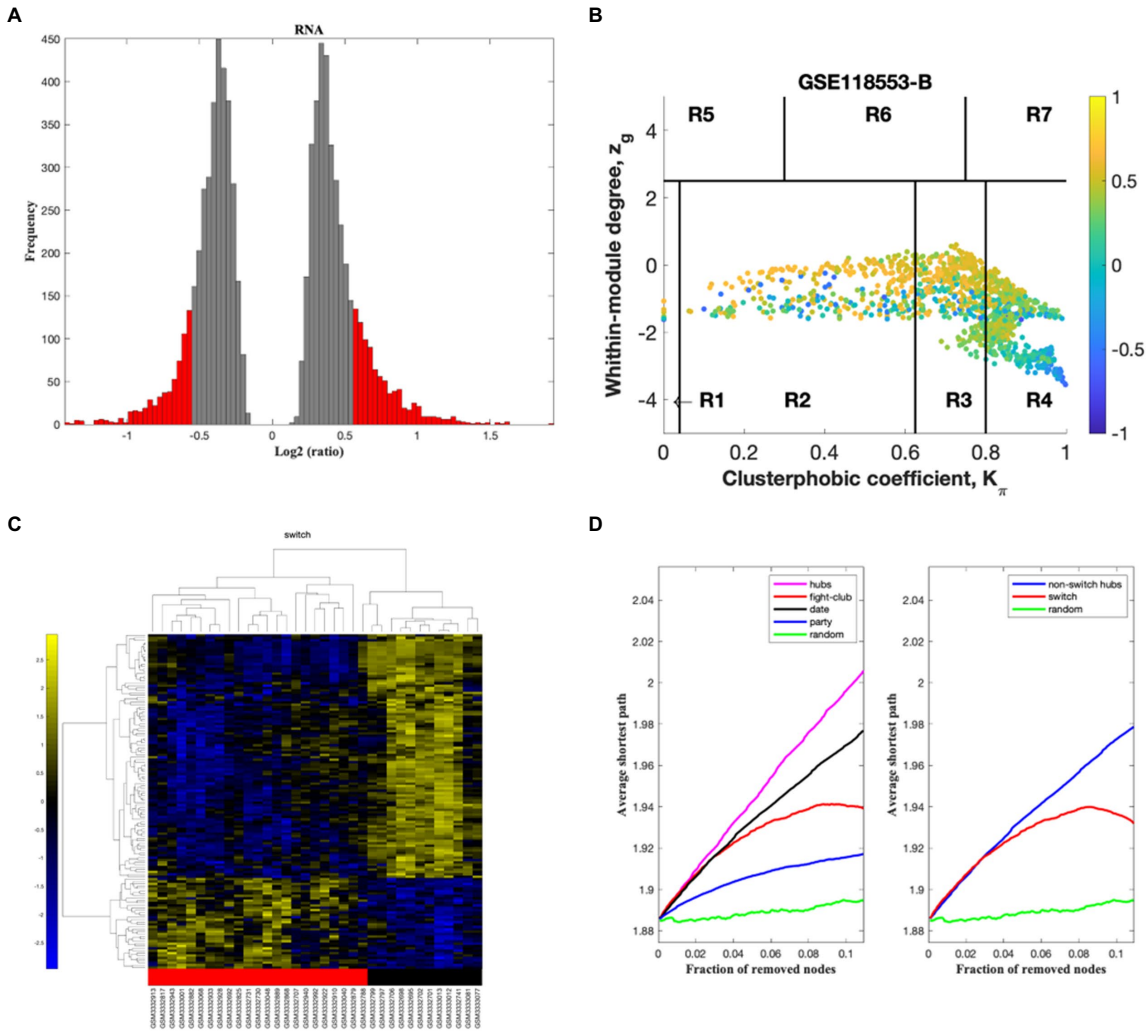
SWIM analysis of entorhinal cortex from female AD compared to normal control subjects in GSE118553. **(A)** Distribution of log2 fold change values where the red bars are selected for further analysis. **(B)** Heat Cartography Map with nodes colored by their average Pearson Correlation Coefficient. Region R4 represents the switch genes. **(C)** Dendrogram and heat map for switch genes. The red markers indicate that the sample came from the diseased cohort. **(D)** Robustness of the correlation network.

The algorithm to identify switch genes consists of several steps. In the initial step, genes are retained (red bars) or eliminated (gray bars) using a cut-off of 1.5 or higher (Figure 2A). We identified the correlation communities in the second step based on the average Pearson correlation coefficient (Figure 2B). The nodes with a negative correlation value with their interaction partner, known as fight club hubs, are shown in R4 in blue (Figure 2B). The parameters *Zg* (within-module degree) and *Kπ* (clusterphobic coefficient) identify the plane, and it is divided into seven regions, each defining a specific node role (R1-R7). High *Zg* values correspond to hubs nodes within their module (local hubs), whereas low *Zg* values correspond to nodes with few connections within their module. Each node is colored according to its average Pearson correlation value. Yellow nodes are party and date hubs, which are positively correlated in expression with their interaction partners. Blue nodes are the fight-club hubs, with an average negative correlation in expression with their interaction partners. The switch genes are denoted by the blue nodes falling in the region R4. Switch genes are characterized by low *Zg* and high *Kπ* values and are connected mainly outside their module. This analysis identified 115, 212, 89, and 122 switch genes from F-AD, F-asymAD, M-AD, and M-asymAD, respectively (Figure 2; Supplementary Table S1). The data presented in Figure 2 corresponds to the analysis of F-AD vs. controls. The analysis of F-AD vs. F-asymAD, M-AD vs. controls, and M-AD vs. M-asymAD is provided in Supplementary Figures S1–S3.

Venn diagram analysis showed that F-AD and F-asymAD shared 15 switch genes, whereas M-AD and M-asymAD shared five switch genes. The unique switch genes were 44, 131, 29, and 69 for F-AD, F-asymAD, M-AD, and M-asymAD, respectively (Supplementary Table S2).

### Biological and functional analysis of switch genes

**Figure 3:**
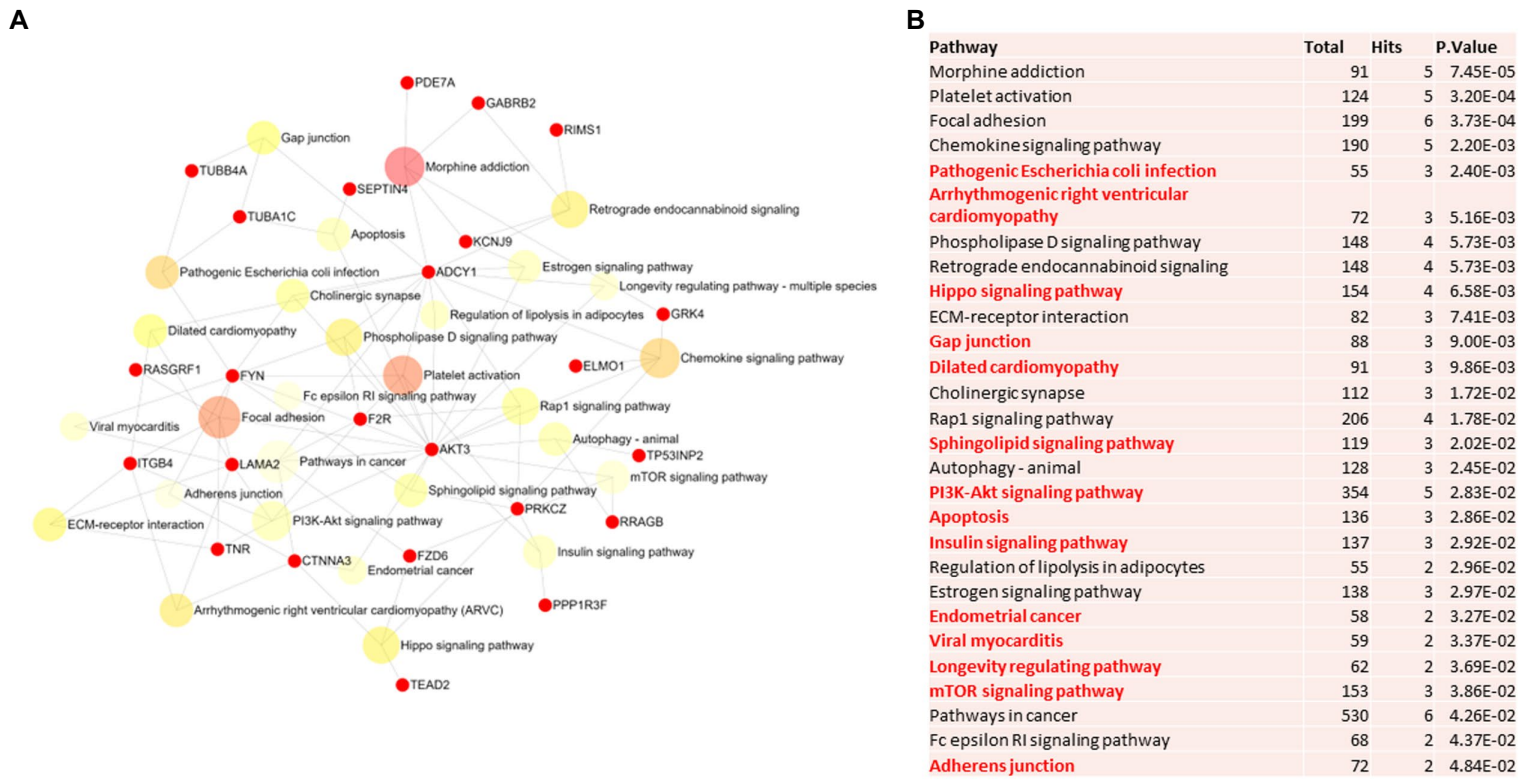
Pathway analysis of switch genes from females with Alzheimer’s disease (F-AD). **(A)** Biological and functional analysis of switch genes from F-AD compared to controls was performed in NetworkAnalyst. Switch genes are depicted in red. **(B)** The pathways are ranked according to the number of hits and lowest value of p. Pathways are derived from the KEGG database. The unique pathways are shown in red.

**Figure 4:**
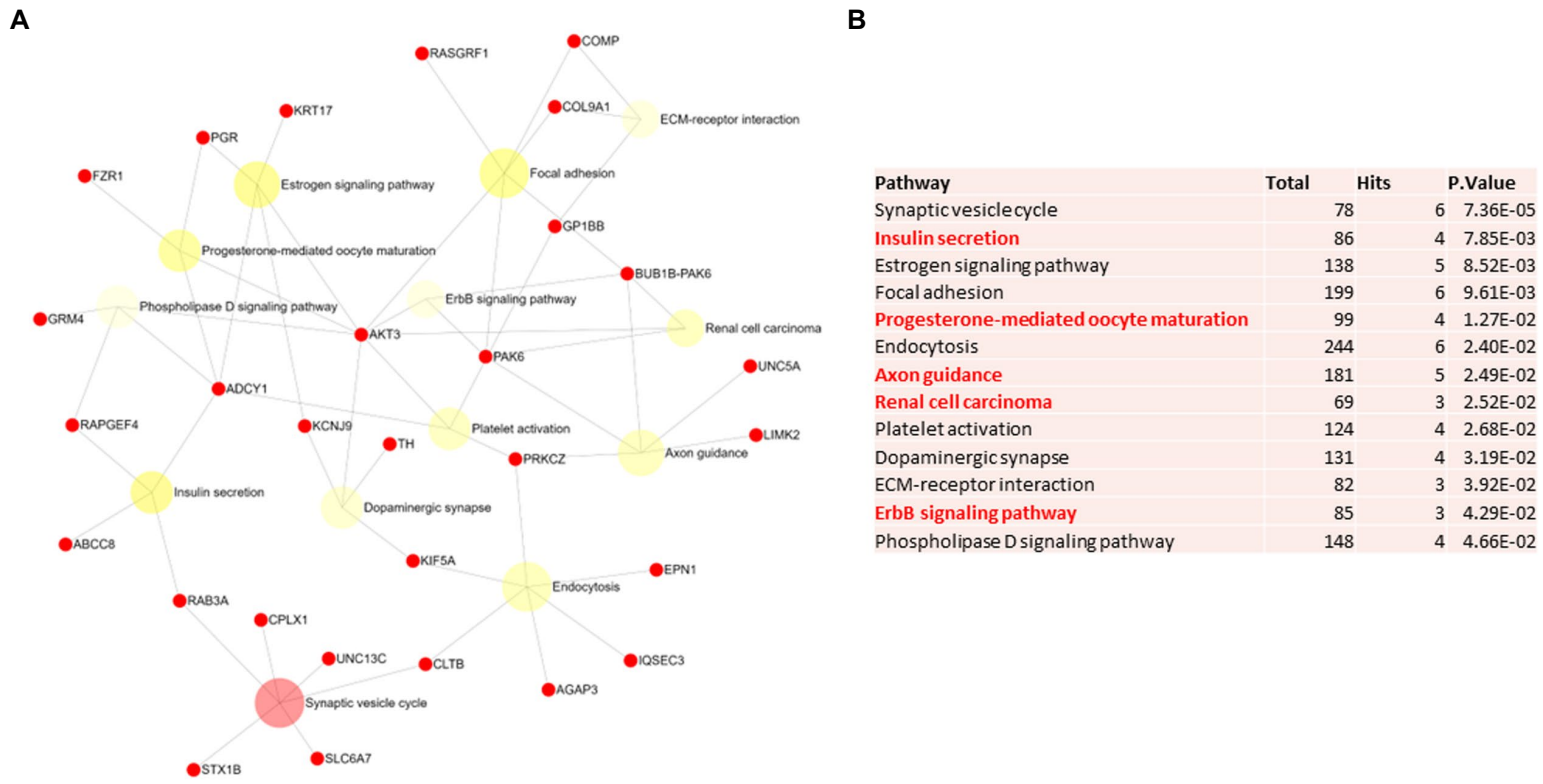
Pathway analysis of switch genes from asymptomatic females with Alzheimer’s disease (F-asymAD). **(A)** Biological and functional analysis of switch genes from F-asymAD compared to controls was performed in NetworkAnalyst. Switch genes are depicted in red. **(B)** The pathways are ranked according to the number of hits and lowest value of p. Pathways are derived from the KEGG database. The unique pathways are shown in red.

**Figure 5:**
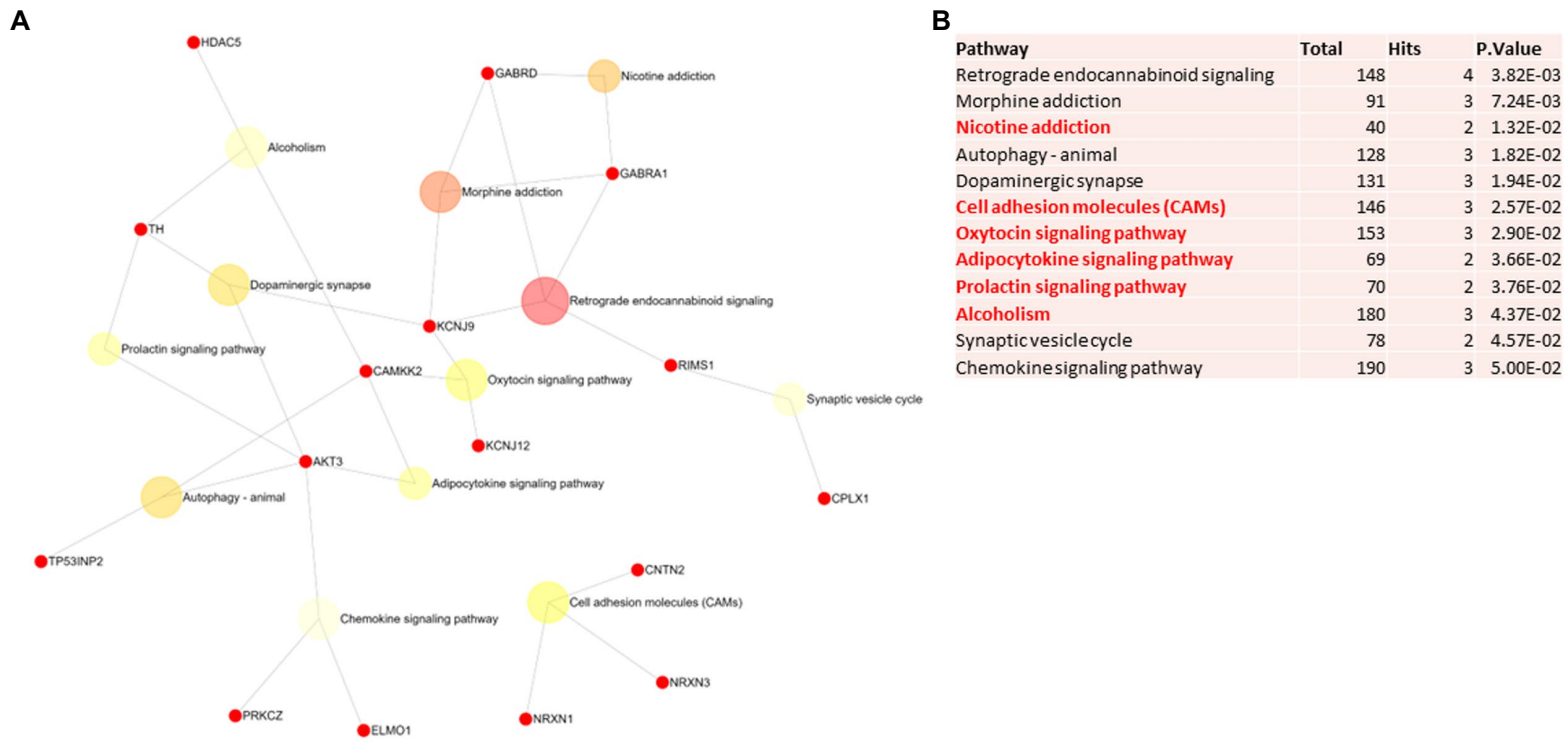
Pathway analysis of switch genes from males with Alzheimer’s disease (M-AD). **(A)** Biological and functional analysis of switch genes from M-AD compared to controls was performed in NetworkAnalyst. Switch genes are depicted in red. **(B)**The pathways are ranked according to the number of hits and lowest value of p. Pathways are derived from the KEGG database. The unique pathways are shown in red.

**Figure 6:**
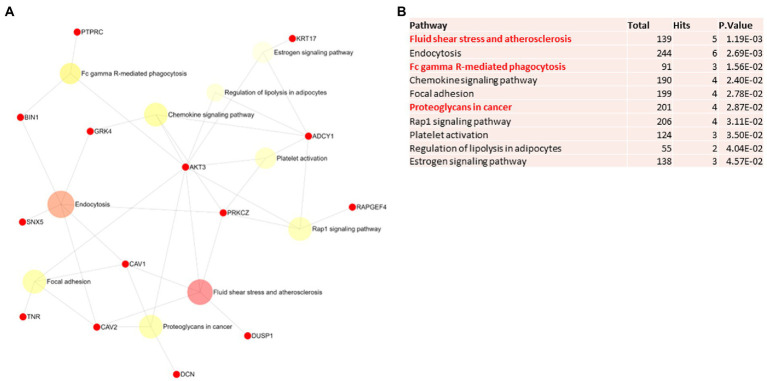
Pathway analysis of switch genes from asymptomatic males with Alzheimer’s disease (M-asymAD). **(A)** Biological and functional analysis of switch genes from M-asymAD compared to controls was performed in NetworkAnalyst. Switch genes are depicted in red. **(B)** The pathways are ranked according to the number of hits and lowest value of p. Pathways are derived from the KEGG database. The unique pathways are shown in red.

Biological and functional analysis of switch genes was performed using NetworkAnalyst. Datasets of switch genes from F-AD, F-asymAD, M-AD, and M-asymAD were analyzed separately (Figures 3A-6A). Functional analysis identified 28, 13, 12, and 10 pathways associated with the switch genes from F-AD, F-asymAD, M-AD, and M-asymAD, respectively. The most significant pathways identified in F-AD switch genes were morphine addiction, platelet activation, and focal adhesion (Figure 3B). The pathways overrepresented in F-asymAD switch genes were synaptic vesicle cycle, insulin secretion, and estrogen signaling (Figure 4B). In contrast, the most significant dysregulated pathways associated with M-AD switch genes were endocannabinoid signaling, morphine addiction, and nicotine addiction (Figure 5B). In contrast, those overrepresented in M-asymAD were related to atherosclerosis, endocytosis, and FcγR mediated phagocytosis (Figure 6B).

We next explored unique pathways associated with each dataset of switch genes. Unique pathways of F-AD switch genes were associated with viral myocarditis, Hippo signaling pathway, endometrial cancer, insulin signaling, PI3K-AKT signaling, adherens junction, gap junction, cardiomyopathy, mTOR signaling, longevity, sphingolipid signaling, apoptosis, *E*. *coli* infection (Figure 3B; Supplementary Table S5). Similarly, unique pathways identified from F-asymAD switch genes were axon guidance, progesterone-mediated oocyte maturation, insulin secretion, renal cell carcinoma, and ErbB signaling (Figure 4B; Supplementary Table S5). In contrast, unique pathways identified from M-AD switch genes associated with alcoholism, nicotine addiction, cell adhesion molecules, prolactin signaling, adipocytokine signaling, and oxytocin signaling (Figure 5B; Supplementary Table S5). Finally, unique pathways from M-asymAD switch genes were atherosclerosis, proteoglycans in cancer, and FcγR mediated phagocytosis (Figure 6B; Supplementary Table S5).

### Transcription factor analysis of switch genes

Transcription factor analysis was performed in NetworkAnalyst using the ENCODE database. The list of transcription factors and Venn diagram analysis are provided in Supplementary Table S3. This analysis yielded 142, 188, 109, and 150 transcription factors associated with the datasets of switch genes from F-AD, F-asymAD, M-AD, and M-asymAD, respectively. Venn diagram analysis revealed that 75 transcription factors were shared among all the groups. F-AD and F-asym AD showed 8 and 30 unique transcription factors, respectively. Similarly, M-AD and M-asymAD showed 1 and 16 unique transcription factors, respectively. Two transcription factors were shared between M-AD and M-asymAD. Thirteen transcription factors were shared between F-AD and F-asymAD.

### Chemicals and drugs interaction analysis of switch genes

In order to investigate potential neuroprotective and neurotoxic agents associated with AD, we performed a protein-chemical and drug network analysis by sex in NetworkAnalyst. The list of chemicals and the results from the Venn diagram analysis are provided in Supplementary Table S4. Switch genes from F-AD, F-asymAD, M-AD, and M-asymAD were analyzed separately. Valproic acid and aflatoxin B were the highest ranked interacting chemicals across all the datasets. Venn diagram analysis identified nine chemicals interacting with F-AD and F-asymAD, including 15-acetyldeoxynivalenol, 2-amino-1-methyl-6-phenylimidazo (4,5-b) pyridine (PhIP), 1,6-hexamethylene diisocyanate, cupric oxide, 4-hydroxy-2-nonenal, tobacco smoke pollution, 1-methyl-4-phenylpyridinium, thapsigargin, and tert-butyl-hydroperoxide. Vincristine was identified as a shared chemical between M-AD and M-asymAD datasets. The F-AD and M-AD datasets shared five chemicals, including 1-butanol, lorazepam, ethanol, MRK 003, and amiodarone.

## Discussion

Numerous AD studies have documented sex-specific differences, yet the molecular mechanisms underlying this association have not been fully characterized. Here we analyzed co-expression networks using SWIM software to investigate sex-specific gene expression changes in the entorhinal cortex of AD patients. The analysis was performed by stratifying symptomatic and asymptomatic AD cases by sex. Co-expression network analysis by SWIM identified unique sex-specific switch genes for F-AD, F-asymAD, M-AD, and M-asymAD groups.

Functional analysis of switch genes revealed several differences in the pathways associated with the switch genes. For instance, morphine addiction, platelet activation, and focal adhesion were the most overrepresented pathways in F-AD. In contrast, in F-asymAD, synaptic vesicle cycle, insulin secretion, and estrogen signaling were the most significant pathways. Though a direct linkage between morphine and AD has not been established, morphine has been associated with AD in several studies. This is not surprising since opioid use is widespread in community-dwelling older adults with and without AD (Bell et al., 2011). However, its use has not been associated with an increased risk of AD (Taipale et al., 2018). Morphine is both neuroprotective and neurotoxic in cellular and animal models of AD. For example, morphine reversed the neurotoxic effects of intracellular amyloid in neuronal cell cultures and rat brains *in vivo* (Cui et al., 2011). Neuroprotection afforded by morphine may be mediated through estradiol release by hippocampal neurons (Cui et al., 2011).

In contrast, morphine has disrupted the homeostasis in neural stem cells by reducing cell growth and expression of insulin-like growth factors and insulin receptors in *in vitro* models (Salarinasab et al., 2017). Given the interaction between opioids with insulin signaling, an association between opioid use and the risk of AD is currently debated (Salarinasab et al., 2020). GABRB2, a switch gene connected to morphine addiction in the F-AD network, has been suggested as a molecular driver of a subtype of AD characterized by amyloid beta neuroinflammation (Neff et al., 2021). Because of the potential association of morphine and insulin signaling, a central pathway in AD pathogenesis, the use of morphine and opioids in AD merits future investigations.

Insulin signaling and diabetes have been extensively implicated in AD and neurodegeneration (Santiago et al., 2019; Santiago and Potashkin, 2021). Interestingly, the results from the pathway analysis revealed that insulin signaling and PI3K-AKT signaling were unique pathways associated with F-AD switch genes. Insulin secretion was one of the most significant and unique pathways in F-asymAD. Diabetes is more prevalent in men than women, with men having twice the odds of having diabetes compared with women (Wild et al., 2004; Nordstrom et al., 2016). Notwithstanding, women with diabetes have a greater risk of developing cardiovascular disease and other complications than men (Kautzky-Willer et al., 2016). A transcriptomic blood analysis revealed that gene expression profiles from women with advanced AD significantly overlapped and correlated negatively with those from diabetes (Santiago et al., 2019). Several switch genes, including AKT3, LAMA2, ADCY1, RAB3A, RAPGEF4, and ABCC8, have been implicated in insulin signaling and diabetes. For example, AKT3, a heavily connected switch gene in the F-AD network, is implicated in insulin signaling and resistance (Sharma and Dey, 2021). AKT3 is downregulated in AD mice treated with a GLP-1 agonist and insulin (Robinson et al., 2019). GLP-1 agonists, drugs commonly prescribed in diabetes, have shown promise in slowing the progression of neurodegenerative diseases (Athauda et al., 2019; Holscher, 2022). Further, AKT3 is associated with microglial inflammation and protection against inflammatory demyelinating disease (DuBois et al., 2019; Ma et al., 2021). Notably, AKT3 plays a vital role in the PI3K-AKT signaling, a potential pathway linking diabetes and AD (Gabbouj et al., 2019; Santiago et al., 2019). Another switch gene, LAMA2, is a serum protein biomarker for pre-diabetes (Yang et al., 2021) and is implicated in high-fat diet-induced obesity (Chen H. J. et al., 2022). ADCY1, RAB3A, and RAPGEF4 switch genes identified in F-asymAD play a key role in pancreatic β-cell insulin secretion (Arora et al., 2012; Kitaguchi et al., 2013; Gucek et al., 2019; Zummo et al., 2022). Mutations in ABCC8 are associated with maturity-onset diabetes of the young (MODY) (Zhang Y. et al., 2022), neonatal diabetes (Lyra et al., 2022), and severe congenital hyperinsulinism (Reyes Diaz et al., 2022). Together, these results suggest that impaired insulin signaling is an important trigger of neurodegeneration among females and may explain the greater prevalence of comorbidities, including cardiovascular disease in females with AD.

Sex hormones have been extensively implicated in sex disparities in AD. Estrogen regulation influences female reproduction and many aspects of brain health including emotions, memory, and cognitive functions (Luine, 2014). Decreased levels of estrogen characterize aging and menopause. This decline in estrogen levels is associated with cognitive impairment and the development of neurodegenerative diseases (Matyi et al., 2019). The role of estrogen in brain health is a subject of ongoing debate. Functional analysis identified estrogen signaling and progesterone-mediated oocyte maturation as some of the most significant pathways associated with F-asymAD switch genes. Epidemiological studies reported a higher incidence of AD in postmenopausal women (Fisher et al., 2018). Endogenous estrogen and hormone replacement therapy correlated positively with higher cognitive status in late life in over 2000 women without dementia (Matyi et al., 2019). Likewise, hormone replacement therapy is associated with a reduced risk of AD in older women (Zandi et al., 2002).

In contrast, estrogen failed to reduce the risk of dementia or cognitive decline but resulted in an increased risk of dementia in older women (Shumaker et al., 2004). Recently, oral contraceptive use and hormone therapy after menopause was associated with a decreased risk of AD in patients with depression (Kim et al., 2022). Several switch genes, including PGR, KRT17, ADCY1, and AKT3 associated with estrogen signaling. KRT17 is a marker of cervical and ovarian cancer (Bai et al., 2019; Di Fiore et al., 2022), and PGR plays a role in estrogen and progesterone signaling (Ikarashi et al., 2021; Hewitt et al., 2022). PGR and ADCY1 have been associated with neurodevelopment, but a direct link with dementia or neurodegeneration has not been found (Siegmund et al., 2007; Sundararajan et al., 2018). The involvement of estrogen-related switch genes in F-AD warrants further mechanistic studies to understand their implications in AD and neurodegeneration better.

In contrast to females, the unique pathways associated with switch genes in M-AD were oxytocin and prolactin signaling, cell adhesion molecules, alcoholism, adipocytokine signaling, and nicotine addiction. The unique pathways identified in M-asymAD were FcγR-mediated phagocytosis, fluid shear stress and atherosclerosis, and proteoglycans. Oxytocin is a neuropeptide hormone that plays a key role in pregnancy by inducing uterine contractions and lactation. In the brain, oxytocin modulates behavior and cognition in several neurological disorders (Guastella et al., 2015; Guastella and Hickie, 2016). Interestingly, oxytocin has been shown to elicit neuroprotection in AD. For instance, intranasal delivery of oxytocin restored cognitive functions in a rodent model of AD (El-Ganainy et al., 2022). Like oxytocin, prolactin is another pituitary hormone associated with immune system regulation, and it has been implicated in various neurological disorders, including AD (Duc Nguyen et al., 2022). Prolactin increases synaptogenesis, axon growth, neuronal plasticity, and memory consolidation (Carretero et al., 2019).

Furthermore, prolactin is associated with inflammatory, anti-inflammatory effects and autoimmunity (Costanza and Pedotti, 2016; Wang et al., 2020). Serum prolactin levels are increased in Huntington’s disease and multiple sclerosis (Duc Nguyen et al., 2022), suggesting it may also be implicated in neurodegeneration. The potential role of oxytocin in AD males warrants a more thorough investigation of hormones in AD.

Alcohol consumption is a subject of extensive debate, with numerous epidemiological studies investigating its association with AD. Excessive alcohol consumption is associated with more significant cognitive decline and lower hippocampal volume in AD patients (Heymann et al., 2016; Zhornitsky et al., 2021). Drinking frequency associated with AD biomarkers in CSF fluid from cognitively intact older individuals (Wang et al., 2021). Chronic alcohol intake induces the generation of reactive oxygen species and hyperglutamatergic excitotoxicity leading to white matter atrophy, axonal loss, demyelination, and neurodegeneration (Kamal et al., 2020). Contrary to these adverse effects, recent studies suggest that low to moderate alcohol consumption could reduce the risk of AD (Andersen et al., 1999; Yang et al., 2022). Several of the M-AD switch genes, HDAC5, TH, and CAMKK2 associated with alcohol. HDAC5 mRNA levels are decreased in the prefrontal cortex of rats sensitized to alcohol and cocaine (Xu S. et al., 2021). Long-term alcohol consumption promotes the degradation of HDAC5 and may increase vulnerability to cocaine addiction (Griffin Jr. et al., 2017). CAMKK2 is involved in ethanol-induced hepatic steatosis, and treatment with caffeic acid, a phytochemical in coffee, increases its mRNA and protein expression, thereby reducing alcohol-mediated damage in mice (Lu et al., 2022). Furthermore, CAMKK2 is associated with amyloid beta-induced neurotoxicity resulting in dendritic spine loss, and its inhibition protected hippocampal neurons against neurotoxicity in a transgenic mouse model of AD (Mairet-Coello et al., 2013).

Cigarette smoking is another important modifiable risk factor in AD. Similar to alcohol, mixed results have been reported on the effects of smoking on AD. For instance, heavy smoking in mid-life is associated with a greater than 100% increased risk of dementia and AD (Rusanen et al., 2011). The mechanisms of smoking-mediated neurodegeneration are unclear, but smoking-associated oxidative stress could exacerbate Aβ pathology (Moreno-Gonzalez et al., 2013). Imaging studies suggest that quitting smoking early in AD could prevent disease progression (Qiu et al., 2022). In contrast, numerous epidemiological and molecular studies indicate that smoking is neuroprotective. Several epidemiological studies reported a lower risk for AD among smokers after controlling for cardiovascular disease, emphysema, and cancer (van Duijn and Hofman, 1991; Brenner et al., 1993). Strikingly, the odds of AD risk increased by 50% every 10 years of smoking cessation (Aggarwal et al., 2006). Among the neuroprotective effects of nicotine identified is the inhibition of Aβ aggregation, protection against NMDA neurotoxicity, and the prevention of neuronal loss by Aβ (Zamani et al., 1997; Aggarwal et al., 2006) reviewed in (Mehta et al., 2012). Several switch genes, including GABRA1 and GABRD, were linked to nicotine addiction in M-AD. A mutation in GABRA1 has been reported in epileptic encephalopathy in children (Chen W. et al., 2022). GABRA1 was identified as a therapeutic target of clinical AD drugs (Aggarwal et al., 2006; Xu Y. et al., 2021). A network co-expression analysis identified GABRD in a key module of genes associated with learning and memory in AD brains (Zhu et al., 2020). The impact of alcohol, smoking, and nicotine addiction on AD is highly debated, and more studies are needed to understand these associations. Identifying switch genes involved in alcohol and nicotine addiction may suggest that men may be more vulnerable to transcriptional changes provoked by alcohol and nicotine than women. Another subject of investigation is whether men are more prone to alcohol or smoking addiction.

Regarding adipocytokine signaling, the release of adipokines by adipose tissue has been shown to play a role in glucose metabolism, lipids, and inflammation Field (Polito et al., 2020), central processes to the pathogenesis of AD. For example, adipokines are directly implicated in obesity and insulin resistance, both risk factors in the pathogenesis of AD (Flores-Cordero et al., 2022). The regulation of these pathways by adipokines may provide neuroprotection from several neurodegenerative diseases, including AD. Adiponectin signaling is involved in the negative regulation of Aβ deposition in preclinical models (He et al., 2021). Similarly, leptin promoted neurogenesis and attenuated Aβ-mediated neurodegeneration in mice (Calio et al., 2021). Impaired leptin signaling is associated with brain structural remodeling changes in obesity and diabetes and thus may play a role in AD [Hayden and Banks, 2021)]. Two switch genes, AKT3 and CAMKK2, were linked to adipocytokine signaling in the M-AD network. Inhibition of CAMKK2 reduces neuronal apoptosis and neuroinflammation in neonatal hypoxic–ischemic encephalopathy and germinal matrix hemorrhage in rodents (Zhang et al., 2018, 2019) and may facilitate the expression of adiponectin, an adipokine that protects against diabetes and atherosclerosis (Kobayashi et al., 2022).

In order to investigate further the functional role of switch genes, we performed a transcription factor analysis. There were eight unique transcription factors associated with the F-AD switch genes. MEF2D is a downstream target of GSK3B associated with neuronal survival in AD (Wang et al., 2009). TSHZ1 regulates pancreatic beta cell maturation and contributes to type 2 diabetes (Raum et al., 2015) and obesity (Berisha et al., 2011). TSHZ1 is also essential for olfactory bulb development and olfaction (Ragancokova et al., 2014). Further, GATA3, a transcription factor crucial in the differentiation of Th2 cells, was identified as a female-specific gene of AD implicated in RNA processing (Eissman et al., 2022).

Analysis of F-asymAD switch genes identified 30 unique transcription factors. Consistent with the pathway analysis, some transcription factors are associated with insulin signaling. For example, CREB3, STAT1, and STAT3 are important regulators of glucose and lipid metabolism in models of high-fat diet and obesity (Bone et al., 2020; Yao et al., 2021; Kiran et al., 2022; Opazo-Rios et al., 2022; Smith et al., 2022). In the context of neurodegeneration, CREB3 and STAT3 are involved in neuroprotective mechanisms. For example, inhibition of STAT3 improved cognition and cerebral blood flow *via* reduction of neuritic plaques, oxidative stress, and neuroinflammation in a rodent model of AD (Mehla et al., 2021). Similarly, regulating STAT1 and STAT3 reduced cognitive dysfunction in a rodent model of AD (Wan et al., 2021). Further, CREB3 contributes to protein degradation in the endoplasmic reticulum (Oh-Hashi et al., 2021) and promotes growth, differentiation, and survival of several neuronal types through stimulation of the nerve growth factor signaling (Sampieri et al., 2021). The results from the transcription factor analysis reinforce the involvement of insulin signaling in females with AD.

In contrast to females, unique transcription factors regulators of switch genes from M-AD and M-asymAD were predominantly associated with cancers. For example, ZHX1, a unique transcription factor in M-AD, has been implicated in gastric cancer, chronic lymphocytic leukemia, and gliomas (Ge and Li, 2020; You et al., 2020; Maciel et al., 2021). TCF7 is involved in prostate cancer (Chen et al., 2015; Siu et al., 2017), and it is highly expressed in immune cells in atherosclerosis plaques (Ma et al., 2022). HMBOX1, a transcription factor involved in innate immunity, showed a strong positive correlation with the Braak score, a measurement of tau pathology severity in AD (Li et al., 2021). Dysregulated expression of NRF1 has been reported in cellular and transgenic animal models of AD (Manczak et al., 2016; Kumar et al., 2019). The findings of transcription factors involved in different cancers may also be linked to the switch genes associated with alcohol and smoking addiction. Unfortunately, information about disease comorbidities was not available for this study. Future consideration of comorbidities and medications will be essential to understand these findings better.

Analysis of chemical and drug interactions with switch genes revealed some sex differences in chemical exposures in AD. For example, nine chemicals including, 15-acetyldeoxynivalenol, 2-amino-1-methyl-6-phenylimidazo(4,5-b)pyridine (PhIP), 1,6-hexamethylene diisocyanate, cupric oxide, 4-hydroxy-2-nonenal, tobacco smoke pollution, 1-methyl-4-phenylpyridinium (MPP+), thapsigargin, and tert-butyl hydroperoxide were identified as interacting with F-AD and F-asymAD. Several of these chemicals have been linked to neurodegeneration. For instance, heterocyclic aromatic amines formed during high-temperature cooking of meats, including PhIP, have been linked to Parkinson’s and Alzheimer’s diseases (Syeda and Cannon, 2022). Exposure to cupric oxide promoted neurotoxicity and neurodegeneration in a *Caenorhabditis elegans* model (Mashock et al., 2016). MPP+ has been extensively studied in the context of neurodegeneration in Parkinson’s disease (Choi et al., 2022; Huo et al., 2022; Zhang J. et al., 2022). Recent evidence suggests that air pollutants and secondhand tobacco smoke are associated with an increased risk of dementia and AD (Peters et al., 2019; Zhou and Wang, 2021). Tert-butyl hydroperoxide triggers oxidative damage and neurotoxicity in neural stem cells (Huang et al., 2018). These results suggest that females may be more vulnerable to these chemicals than males. To the best of our knowledge, sex differences in the exposure to these chemicals have not been studied.

In males, vincristine was the only interacting drug shared between M-AD and M-asymAD switch genes. Vincristine is a chemotherapeutic drug for several cancers known to cause motor neurotoxicity and neuropathies (van de Velde et al., 2021; Dakik et al., 2022). The finding of a chemotherapeutic may also be related to the cancer-associated pathways and transcription factors regulating the switch genes identified in males.

Several limitations should be considered when interpreting the results of this study. As noted in the original study, the asymptomatic group of AD subjects may consist of a heterogeneous group of cognitively normal, mild cognitive impairment, and mixed dementias. These asymptomatic subjects may develop AD or maybe be resilient to the disease. Notably, the results presented in this study are entirely based on bioinformatics methods of publicly available data. In addition, there are fewer males than females represented in this study. Also, there is no information about disease comorbidities or medications in the original study. Comorbidities and medication usage are potential confounding factors that need further investigation. Future molecular and mechanistic studies will be required to confirm the functional role of these genes in driving sex differences in AD.

## Data availability statement

The original contributions presented in the study are included in the article/Supplementary Material, further inquiries can be directed to the corresponding author.

## Ethics statement

Ethical review and approval was not required for the study on human participants in accordance with the local legislation and institutional requirements. The patients/participants provided their written informed consent to participate in this study.

## Author contributions

JS, JQ, and JP conceived and designed experiments. JS and JQ performed the experiments. JS and JP wrote the manuscript. JP acquired funding. All authors have read and approved the final version of the manuscript.

## Funding

This study was funded by the National Institute on Aging (NIA) grant number R01AG062176 to JP. In addition, funds were provided by Rosalind Franklin University of Medicine and Science.

## Conflict of interest

JS is the founder of NeuroHub Analytics, LLC. JQ is the founder of Q Regulating Systems, LLC.

The remaining author declares that the research was conducted in the absence of any commercial or financial relationships that could be construed as a potential conflict of interest.

## Publisher’s note

All claims expressed in this article are solely those of the authors and do not necessarily represent those of their affiliated organizations, or those of the publisher, the editors and the reviewers. Any product that may be evaluated in this article, or claim that may be made by its manufacturer, is not guaranteed or endorsed by the publisher.

## Supplementary material

The Supplementary material for this article can be found online at: https://www.frontiersin.org/articles/10.3389/fnagi.2022.1009368/full#supplementary-material

## Footnotes

^1^ https://www.networkanalyst.ca/
