# Supplementary figures and images for: Sex-specific transcriptional rewiring in the brain of Alzheimer’s disease patients
Source: Front Aging Neurosci. 2022 Oct 31;14:1009368. doi: 10.3389/fnagi.2022.1009368 (PMC9659968; doi:10.3389/fnagi.2022.1009368)

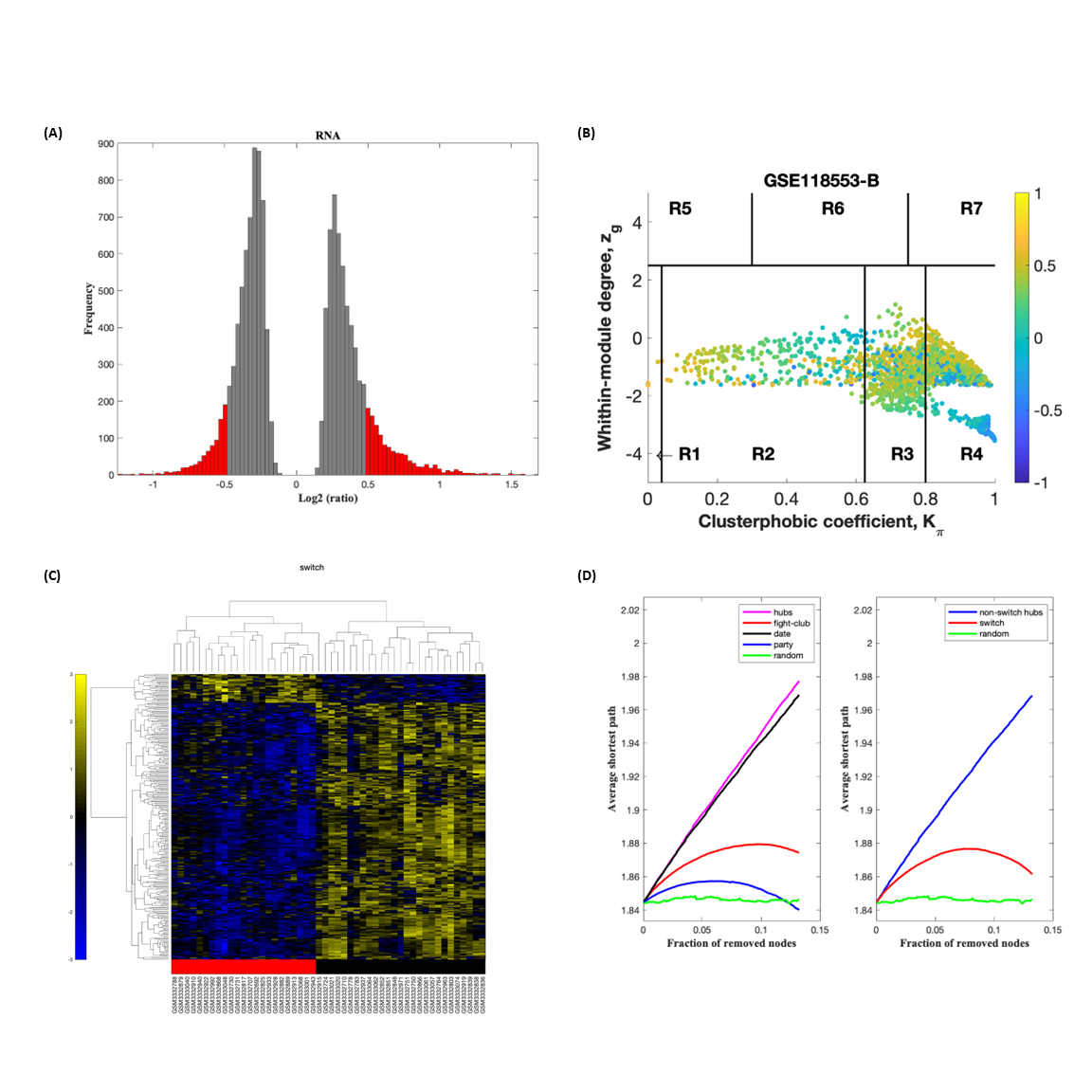

Supplement: Supplementary file 6 [file Image_1.TIF]

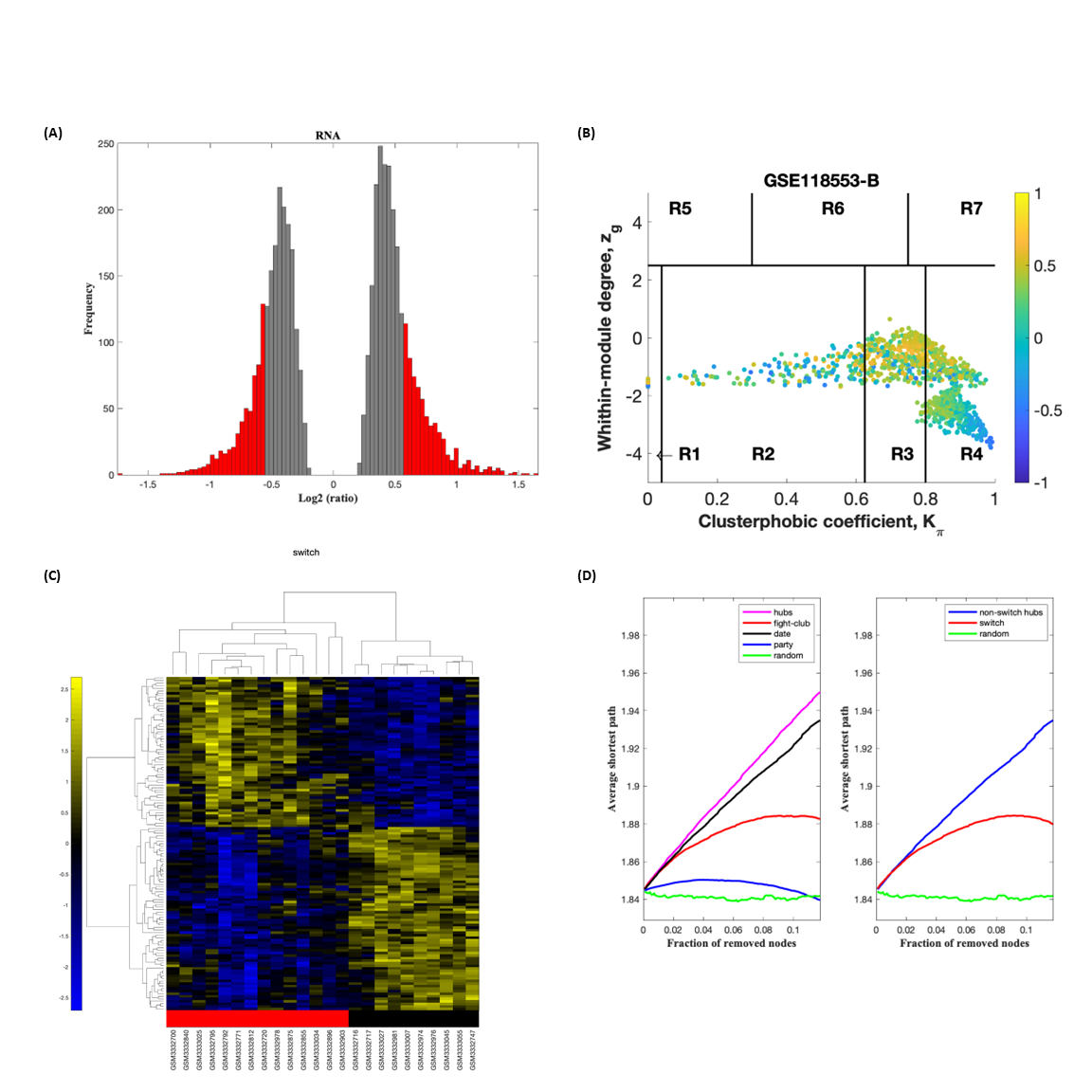

Supplement: Supplementary file 8 [file Image_3.TIF]
